# Supplementary material for: Deciphering and integrating invariants for neural operator learning with various physical mechanisms
Source: Natl Sci Rev. 2023 Dec 29;11(4):nwad336. doi: 10.1093/nsr/nwad336 (PMC10939376; doi:10.1093/nsr/nwad336)
Supplement: nwad336_Supplemental_File [file nwad336_supplemental_file.pdf]

Supplementary Materials for
Deciphering and integrating invariants for neural operator learning with various physical mechanisms

Rui Zhang[1], Qi Meng[2*], and Zhi-Ming Ma[1*]

[1]Academy of Mathematics and Systems Science, Chinese Academy of Sciences (CAS), Beijing 100190, China

[2]Microsoft Research, Beijing 100080, China

[*]**Corresponding author.** Email: meq@microsoft.com, mazm@amt.ac.cn

Contents

1 EXPERIMENTAL DETAILS **2**
1.1 Examples of the dataset . . . 2
1.2 Baselines . . . 2
1.3 Training details . . . 2
1.4 Computational costs . . . 3

2 ADDITIONAL ANALYSES FOR PI ENCODER **3**
2.1 The ablation studies on PI encoder . . . 4
2.2 The effects of hyper-parameters in contrastive training . . . 4
2.3 OOD generalization ability of PI encoder . . . 5
2.4 PI in the real-world scenario . . . 5

1 EXPERIMENTAL DETAILS

In this section, we introduce the experimental details in the main body.

1.1 Examples of the dataset

Examples of each dataset can be seen in Supplementary Fig. 1.

1.2 Baselines

We consider several representative baselines from operator learning models, including:

- Fourier Neural Operator (FNO) [5]: A classical neural operator that uses the Fourier transform to handle PDE information in the frequency domain. The number of layers for FNO is fixed at 4, and we set the modes of the Fourier layer at 16 and 12 for the 1D and 2D cases, respectively, which is selected from the set $\{12, 16, 20\}$.
- Unet [6, 7]: A classic architecture for semantic segmentation in biomedical imaging, recently utilized as a surrogate model for PDE solvers. The architecture of Unet is in line with the PDE benchmark paper [7], and we adjust the width of the convolutional layers to make its size align with other baselines.
- Low-rank decomposition Network (LordNet) [4]: A convolutional-based neural PDE solver that learns a low-rank decomposition layer to extract dominant patterns. The architecture of LordNet is in line with its original paper, and we adjust the width of the convolutional layers to make its size align with other baselines.
- MultiWaveleT-based model (MWT) [2]: A neural operator that compresses the kernel of the corresponding operator using a fine-grained wavelet transform. The architecture of MWT is in line with its original paper, and we select the parameter α in the MultiWavelet layer from the set $\{16, 18, 20\}$.
- Factorized Fourier Neural Operators (FFNO) [8]: A FNO variant that improves performance using a separable spectral layer and enhanced residual connections. Since the codes for FFNO are only available for 2D cases, we only conduct it on 2D NSE data. The number of layers for FFNO is fixed at 6, and we fix the modes of the Fourier layer at 12 for 2D cases, respectively, which is selected from the set $\{12, 16, 20\}$.

For PIANO, we conduct experiments on PIANO + X, where X represents the backbone models. For the neural operator X and PIANO + X, we align the critical parameters of X (e.g., modes in FNO and FFNO, number of layers in neural networks) and adjust the widths of the networks to match the number of parameters between X and PIANO + X, thereby ensuring a fair comparison. These critical parameters in the baseline methods are chosen with the help of validation set. In 1D cases, we choose FNO and Unet as backbone models for PIANO and additionally incorporate FFNO for 2D cases. The PI encoder in PIANO is a much lighter network compared to its backbone model. For Burgers' data, the PI encoder is stacked with two Fourier layers ($\mathrm{modes} = 10$, $\mathrm{width} = 10$), two convolutional layers ($\mathrm{width} = 10, \mathrm{kernel\_size} = 4$) and a two-layer MLP. For CDE data, the PI encoder is stacked with two Fourier layers ($\mathrm{modes} = 8, \mathrm{width} = 16$), two convolutional layers ($\mathrm{width} = 10, \mathrm{kernel\_size} = 4$), and a two-layer MLP. For NSE, the PI encoder is stacked with two Fourier layers ($\mathrm{modes} = 10, \mathrm{width} = 12$), two convolutional layers ($\mathrm{width} = 12, \mathrm{kernel\_size} = 4$) and a two-layer MLP. For the backbone model in PIANO, we modify its first layer to the DyConv layer and the number of DyConv layers K is fixed at 4 for all tasks.

1.3 Training details

For each method, we employ the AdamW optimizer to train the neural network for $20,000$ epochs, using an initial learning rate of lr. The learning rate is decayed by 0.8 every $5,000$ epoch. The optimal initial learning rate lr is chosen from the set $\{0.001, 0.002, 0.005, 0.01\}$ for each method with the help of validation set. A fixed batch size of 200 is used throughout the training process. In the training stage, we evaluate the model's performance on the validation data every 20 epoch and apply early stopping to the optimization if the model's performance does not improve in $2,000$ iterations. During the contrastive

Supplementary Table 1: **Computational costs of PIANO and its corresponding backbone model.** The training of PIANO consists of two parts: the PI encoder (PE) and the neural operator (NO). During the inference stage, PIANO first encodes the initial field of the PDE into its corresponding PI embedding, and calculates the attention (ATT) to obtain a personalized neural operator (PNO). After that, PIANO predicts the subsequent PDE field using this personalized neural operator.

Data	Model	Training Time (s)			Inference Time (s)				Model Size (M)			
		PE	NO	Total	PE	ATT	NO	Total	PE	NO	Total	PNO
**E1**	FNO	–	0.128	0.128	–	–	0.018	0.018	–	–	0.757	–
	PIANO + FNO	0.029	0.118	0.147	0.002	0.004	0.016	0.022	0.053	0.709	0.762	0.553
**E2**	FNO	–	0.384	0.384	–	–	0.182	0.182	–	–	2.085	–
	PIANO + FNO	0.067	0.328	0.395	0.004	0.011	0.123	0.138	0.184	1.836	2.020	1.024
	FFNO	–	1.964	1.964	–	–	1.008	1.008	–	–	2.013	–
	PIANO + FFNO	0.067	1.297	1.364	0.004	0.010	0.668	0.682	0.184	1.704	1.888	1.136

training stage in PIANO, we also use the AdamW optimizer to train the neural network for $20,000$ epochs, with an initial learning rate of $\mathrm{lr} = 0.001$ and a learning rate decay factor of 0.5 every $4,000$ epochs. Batch size and temperature parameter τ are maintained at 512 and 0.5, respectively. Please note that the PIs in E1-E6 correspond to different PIs, including spatiotemporal invariant, boundary invariant, and temporal invariant, respectively. Therefore, we need to utilize the corresponding cropping strategies. Moreover, we need to train two PI encoders for E5 and combine the corresponding PI embeddings because there are two types of invariants in the settings. When conducting the split-merge trick, we set the spatial resolution to 32 for 1D scenarios and 32×32 for 2D scenarios, respectively. In the SimCLR settings, the dimensions of $\boldsymbol{h}$ and $\boldsymbol{z}$ are set as 64 and 32, respectively.

1.4 Computational costs

We present the computational cost in the training and inference stages for PIANO and its corresponding backbone models in Supplementary Table 1. We summarize our observations as follows:

- The training costs for the PI encoder are relatively low compared to the training costs of the neural operator. The reason is that we use the split-merge trick to divide the physics fields into smaller patches (e.g., from 64×64 to 32×32 in E2), and the model size of the PI encoder is much lighter compared to the neural operator.
- During the inference stage, PIANO first encodes the initial field of the PDE into its corresponding PI embedding and calculates the attention to obtain a personalized neural operator. Given the small size of the PI encoder and the fact that attention in the DyConv only involves weighted summation calculations, these computations are lightweight. As we have aligned the model size of PIANO + X with X, the scale of the personalized neural operator is significantly smaller than that of the original operator X. Therefore, it can predict subsequent PDE fields faster once the personalized operator is determined.
- In the 1D case, due to the inherent speed of the neural operator, the inference speeds of PIANO and PIANO + X are similar. However, in 2D cases, inference speed is significantly different between neural operators of different scales. Therefore, the benefits brought by PIANO become more apparent.

2 ADDITIONAL ANALYSES FOR PI ENCODER

In this section, we conduct additional experiments to examine the effects and properties of the PI encoder in PIANO. First, we perform ablation studies to uncover the impact of specific technical aspects of the PI encoder. Next, we study the effects of two hyperparameters, including $\mathrm{batch\_size}$ and the temperature τ in contrastive learning for PIANO. Finally, we investigate the out-of-distribution (OOD) generalization ability of the PI encoder and the its performance in the real-world scenario.

Supplementary Table 2: **Results of ablation studies on Burgers' and NSE data.** Relative ℓ_2 errors (E_{ℓ_2}, %) for baseline methods and PIANO on training and future domains. CL, SM, and PC are the abbreviations of contrastive learning, split-merge trick, and physics-aware cropping strategy, respectively. The best results in each task are in **bold**.

Data	Model	Training Domain	Future Domain
Burgers'	FNO	0.668 $\pm$ 0.039	1.062 $\pm$ 0.039
	PIANO-~~CL~~	0.782 $\pm$ 0.010	1.253 $\pm$ 0.030
	PIANO-~~SM~~	0.662 $\pm$ 0.021	0.845 $\pm$ 0.062
	PIANO-~~PC~~	0.691 $\pm$ 0.046	0.884 $\pm$ 0.080
	PIANO	**0.492 $\pm$ 0.045**	**0.536 $\pm$ 0.046**
NSE	FNO	10.433 $\pm$ 0.298	30.702 $\pm$ 1.043
	PIANO-~~CL~~	11.978 $\pm$ 0.733	35.543 $\pm$ 3.938
	PIANO-~~SM~~	5.566 $\pm$ 0.328	22.196 $\pm$ 1.148
	PIANO-~~PC~~	5.882 $\pm$ 0.328	22.881 $\pm$ 0.978
	PIANO	**4.652 $\pm$ 0.396**	**17.393 $\pm$ 0.672**

2.1 The ablation studies on PI encoder

In this section, we conduct ablation studies to demonstrate the effects of essential components within the PI encoder for PDE forecasting tasks on Burgers' (E1) and NSE (E4) data. Revisiting the training stages of the PI encoder, contrastive learning plays an essential role in extracting the underlying PIs for PDE systems. Additionally, we utilize some technical tricks to enhance the performance of PI encoders, including the split-merge trick and the physics-aware cropping strategy. To evaluate the effects of each component in PIANO, we compare several corresponding baselines as follows:

- PIANO-~~CL~~: In this model, we jointly train the PI encoder and neural operator without the contrastive pre-training, which can be regarded as an FNO version for DyConv. We train this model to reveal the impact of contrastive learning in PIANO.
- PIANO-~~SM~~: In PIANO, we utilize the split-merge trick to divide the PDE fields Ω into several patches $\{\Omega_v\}_{v=1}^{V}$ and then input them into the PI encoder during both training and testing phases. In PIANO-~~SM~~, we directly feed the entire PDE fields into the PI encoder, i.e., utilize the global cropping method.
- PIANO-~~PC~~: We assert that the cropping strategies should align with the physical prior of the PDE system and propose the physics-aware cropping methods for contrastive learning. In PIANO-~~PC~~, we discard the physics-aware cropping technique and swap two corresponding augmentation methods for Burgers' and NSE data, respectively.

Based on the ablation experiment results for Burgers' and NSE data (Supplementary Table 2), the performance of PIANO-~~CL~~ is even worse than that of the plain FNO, which highlights the crucial role of contrastive learning in PIANO. Moreover, two distinct variations of PIANO, namely PIANO-~~SM~~ and PIANO-~~PC~~, demonstrate inferior performance in comparison to the original PIANO model, which indicates that we need to consider the properties of PIs and make the most of them during the training stages of PI encoder.

2.2 The effects of hyper-parameters in contrastive training

According to the existing SimCLR experiences reported in [1], two hyperparameters, $\mathrm{batch\_size}$ and the temperature τ, have a significant impact on the quality of the representations. In this section, we study the effects of these two hyperparameters on the quality of PI representations. Firstly, we fix the hyperparameter τ to 0.5 and train the PI encoders with the $\mathrm{batch\_size}$ values from the set $\{64, 128, 256, 512\}$. Similarly, we fix the hyperparameter $\mathrm{batch\_size}$ to 512 and train the PI encoders with the τ values of the set $\{0.01, 0.05, 0.1, 0.5\}$. Supplementary Table 3 presents the effects of hyperparameters on the quality of PI representations in contrastive learning using linear evaluation. The performance of the PI encoder remains relatively stable with changes in $\mathrm{batch\_size}$ for both Burgers' equation and NSE, particularly when $\mathrm{batch\_size}$ is greater than 256. Regarding the temperature parameter τ, the performance of the

Supplementary Table 3: **The effects of hyper-parameters on the quality of the PI representations in contrastive learning.** Accuracy (relative ℓ_2 error) of the PI encoder trained with varying $\mathrm{batch\_size}$ and τ using linear evaluation on Burgers' equation (NSE). The best results in each task are in **bold**.

The effects of $\mathrm{batch\_size}$				
$\mathrm{batch\_size}$	64	128	256	512
Burgers' (Acc., ↑)	0.985± 0.009	0.985± 0.009	0.993± 0.003	**0.997**± **0.003**
NSE (ℓ_2 Error, ↓)	0.035± 0.002	0.033± 0.002	**0.032**± **0.001**	0.033± 0.002
The effects of τ				
τ	0.01	0.05	0.1	0.5
Burgers' (Acc., ↑)	0.987± 0.008	0.985± 0.009	0.988± 0.003	**0.997**± **0.003**
NSE (ℓ_2 Error, ↓)	0.045± 0.007	0.051± 0.006	0.048± 0.006	**0.033**± **0.002**

PI encoder for both Burgers' equation and NSE generally improves as it increases, suggesting that a relatively larger τ is recommended for training the PI encoder with SimCLR in PIANO.

2.3 OOD generalization ability of PI encoder

In this section, we conduct experiments to evaluate the out-of-distribution (OOD) generalization ability of the PI encoders in PIANO on E1 and E4. In E1, we add two groups of PDE data driven by external forcing $\cos(4x)$ and $\sin(4x)$. In E2, we add fluid fields with viscosity terms ranging from $[10^{-2}, 10^{-1}]$. Please note that the PI corresponding to these newly added data has never appeared in the training set. We use UMAP to map the PI embeddings to a low-dimensional space and show the results in Supplementary Fig. 2. It is interesting to see that the PI encoder can distinguish PDE fields outside the distribution. However, according to our experiments, PIANO fails to obtain meaningful forecasting results on PDE simulation tasks on out-of-distribution data. For instance, the relative error in E1 on the OOD data is larger than 40%, while the one on in-distribution data is less than 0.5%. One potential reason is that forecasting is much more challenging than learning PI, so data may be indispensable. One promising direction is to leverage few-shot learning techniques to transfer PIANO to other distributions [9], which we leave as an important future work.

2.4 PI in the real-world scenario

In this section, we train the PI encoder on weather forecasting data [3] to demonstrate our assumptions and motivations in real-world scenarios. When training the PI encoder, we utilize the prior knowledge that geographical information such as altitude and coastline does not change over time. Therefore, we regard them as spatial invariants and use the corresponding cropping techniques in the contrastive learning stage.

In detail, we consider two kinds of weather data in this section, including the 2m temperature for the mainland and the sea surface temperature data. The range of mainland data is from 15 degrees north latitude to 75 degrees north latitude, and from 60 degrees east longitude to 120 degrees east longitude. The range of sea data is from 60 degrees south latitude to the equator, and from 150 degrees west longitude to 90 degrees west longitude. The series in the dataset is randomly sampled from the temperature data of the full year from 2020 to 2022.

To demonstrate that the trained PI embedding indeed contains geographical information, we select six different regions from each dataset and cut 200 series corresponding to different times from each region. Each series has a spatial resolution of 64×64, with each grid point representing the average temperature at a location of 0.25° longitude and 0.25° latitude. Each series has 168 frames, with an interval of two hours between each frame. We use UMAP to reduce the dimensionality of these 1200 series' PI embeddings to a 2D low-dimensional space for land and sea datasets, respectively. The dimensionality reduction results in Supplementary Fig. 3 indicate that the PI encoder can distinguish different geographical regions, i.e., it has learned the corresponding geographical information.

References

[1] Ting Chen, Simon Kornblith, Mohammad Norouzi, and Geoffrey E. Hinton. A simple framework for contrastive learning of visual representations. In *Proceedings of the 37th International Conference on Machine Learning, ICML 2020, 13-18 July 2020, Virtual Event*, volume 119 of *Proceedings of Machine Learning Research*, pages 1597–1607, 2020.

[2] Gaurav Gupta, Xiongye Xiao, and Paul Bogdan. Multiwavelet-based operator learning for differential equations. *Advances in neural information processing systems*, 34:24048–24062, 2021.

[3] Hans Hersbach, Bill Bell, Paul Berrisford, Gionata Biavati, András Horányi, Joaquín Muñoz Sabater, Julien Nicolas, Carole Peubey, Raluca Radu, Iryna Rozum, et al. Era5 hourly data on single levels from 1979 to present. *Copernicus climate change service (c3s) climate data store (cds)*, 10(10.24381), 2018.

[4] Xinquan Huang, Wenlei Shi, Qi Meng, Yue Wang, Xiaotian Gao, Jia Zhang, and Tie-Yan Liu. Neuralstagger: Accelerating physics-constrained neural PDE solver with spatial-temporal decomposition. In *International Conference on Machine Learning, ICML 2023, 23-29 July 2023, Honolulu, Hawaii, USA*, volume 202 of *Proceedings of Machine Learning Research*, pages 13993–14006, 2023.

[5] Zongyi Li, Nikola Borislavov Kovachki, Kamyar Azizzadenesheli, Burigede Liu, Kaushik Bhattacharya, Andrew M. Stuart, and Anima Anandkumar. Fourier neural operator for parametric partial differential equations. In *9th International Conference on Learning Representations, ICLR 2021, Virtual Event, Austria, May 3-7, 2021*, pages 1–16, 2021.

[6] Olaf Ronneberger, Philipp Fischer, and Thomas Brox. U-net: Convolutional networks for biomedical image segmentation. In *Medical Image Computing and Computer-Assisted Intervention–MICCAI 2015: 18th International Conference, Munich, Germany, October 5-9, 2015, Proceedings, Part III 18*, pages 234–241, 2015.

[7] Makoto Takamoto, Timothy Praditia, Raphael Leiteritz, Daniel MacKinlay, Francesco Alesiani, Dirk Pflüger, and Mathias Niepert. Pdebench: An extensive benchmark for scientific machine learning. In *Annual Conference on Neural Information Processing Systems 2022, NeurIPS 2022, December 12-16, 2022, New Orleans, Louisiana, USA*, pages 1–16, 2022.

[8] Alasdair Tran, Alexander Patrick Mathews, Lexing Xie, and Cheng Soon Ong. Factorized fourier neural operators. In *The Eleventh International Conference on Learning Representations, ICLR 2023, Kigali, Rwanda, May 1-5, 2023*, pages 1–17, 2023.

[9] Yaqing Wang, Quanming Yao, James T Kwok, and Lionel M Ni. Generalizing from a few examples: A survey on few-shot learning. *ACM computing surveys (csur)*, 53(3):1–34, 2020.

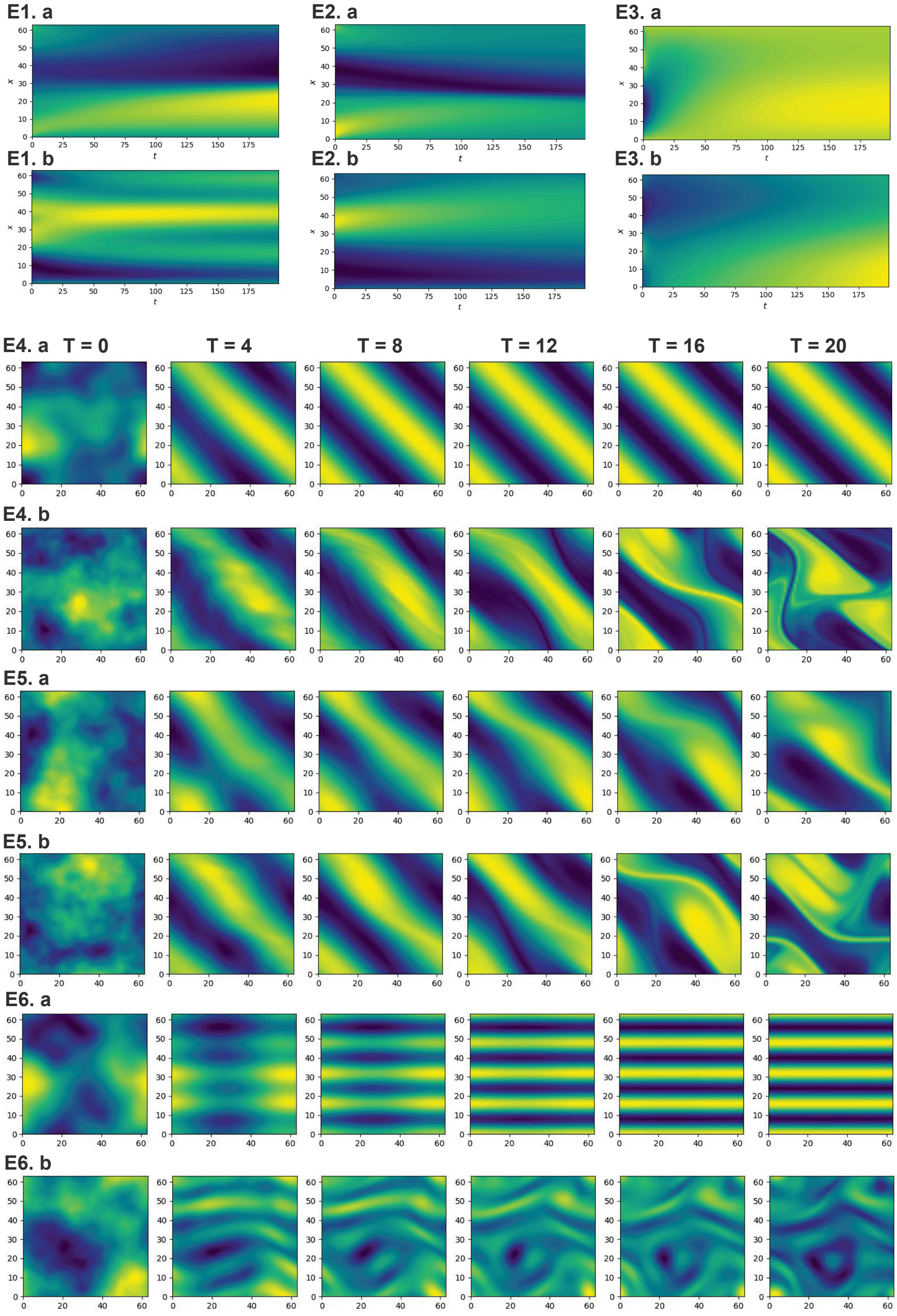

Supplementary Figure 1: **Examples in the dataset of E1, E2, E3, E4, E5 and E6.** **E1** (Burgers' Equation with varying external forces f). External forces $f(x)$ are set as $-\tanh(3x)$ and $\sin(3x)$ in E1.a and E1.b, respectively. **E2** (Burgers' Equation with varying diffusivies D). The diffusivies $D(x)$ are set as $1 - \cos(x)$ and $1 + \sin(x)$ in E2.a and E2.b, respectively. **E3** (CDE with varying boundary conditions $\mathcal{B}$). The boundary conditions are set as Dirichlet and Robin in E3.a and E3.b, respectively. **E4** (NSE with varying viscosity terms ν). The viscosity terms ν are set as 10^{-2} and 10^{-5} in E4.a and E4.b, respectively. **E5** (NSE with varying viscosity terms ν and external forces f). The viscosity terms ν and the coefficients in f are set as (4×10^{-4}, 0.091) and (10^{-4}, 0.135) in E5.a and E5.b, respectively. **E6** (Kolmogorov flow with varying viscosity terms ν). The viscosity terms ν are set as 10^{-2} and 10^{-4} in E6.a and E6.b, respectively.

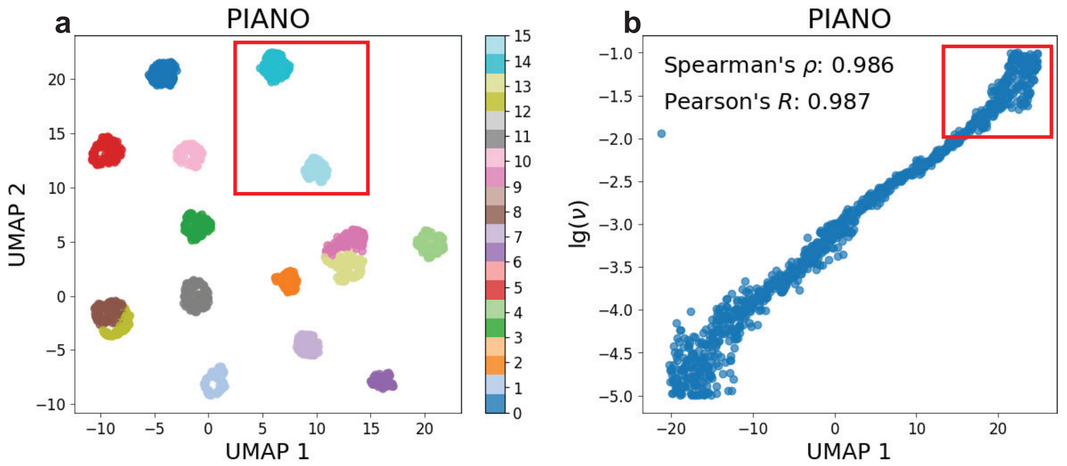

Supplementary Figure 2: **The out-of-distribution (OOD) generalization ability of the PI encoder. a:** The dimensionality reduction results of PI embeddings via UMAP for Burgers' data (E1) are shown. The horizontal and vertical axes represent the two main components of UMAP, and each color represents a different external force in the dataset. The red box encloses the PDE fields driven by the forcing $\sin(4x)$ and $\cos(4x)$, which have never appeared in the training set. **b:** The dimensionality reduction results of the PI embeddings via UMAP for the NSE data (E4) are presented. The horizontal and vertical axes represent the first component of UMAP and the logarithmic viscosity term $\lg(\nu)$ in the dataset. The red box encloses the NSE fields with viscosity terms ranging from 10^{-1} to 10^{-2}, which have never appeared in the training set.

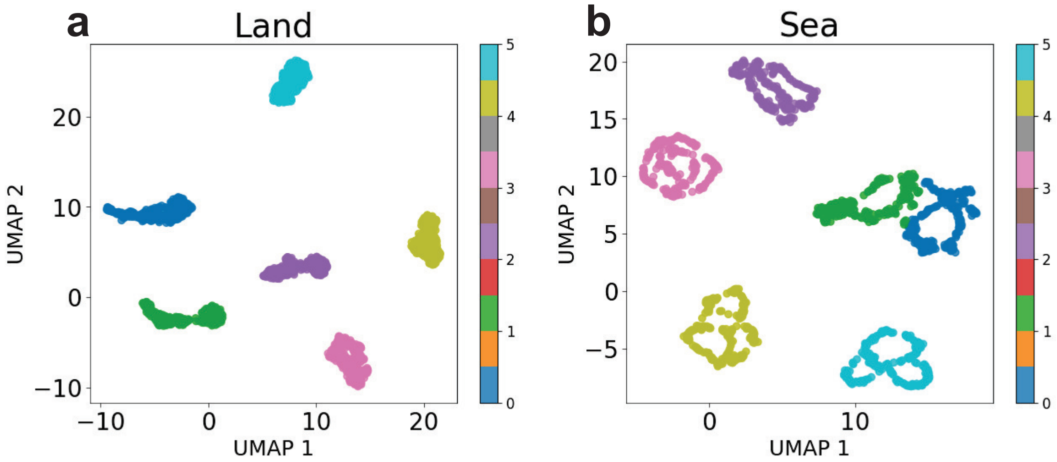

Supplementary Figure 3: **The performance of the PI encoder in the real-world scenario. a and b:** The dimensionality reduction results of PI embeddings via UMAP for land and sea temperature data, respectively. The horizontal and vertical axes represent the two main components of UMAP, and each color represents a different spatial region in the dataset.
